# Peri- and postoperative morbidity and mortality in older patients with non-small cell lung cancer: a matched-pair study

**DOI:** 10.1186/s12957-024-03491-6

**Published:** 2024-08-08

**Authors:** Seyer Safi, Maximilian Robert Gysan, Dorothea Weber, Rouven Behnisch, Thomas Muley, Michael Allgäuer, Hauke Winter, Hans Hoffmann, Martin Eichhorn

**Affiliations:** 1 Division of Thoracic Surgery, University Hospital rechts der Isar, Technical University of Munich, Munich, Germany; 2 Department of Thoracic Surgery, Heidelberg University Hospital, Thoraxklinik, Heidelberg, Germany; 3 Institute of Medical Biometry and Informatics, University of Heidelberg, Heidelberg, Germany; 4 Translational Lung Research Center Heidelberg (TLRCH), Member of the German Center for Lung Research (DZL), Heidelberg, Germany; 5 Translational Research Unit, Heidelberg University, Thoraxklinik, Heidelberg, Germany; 6 Institute of Pathology, Heidelberg University Hospital, Heidelberg, Germany; 7 Division of Pulmonology, Department of Internal Medicine II, Medical University of Vienna, Vienna, Austria

## Abstract

**Background:**

Reports from case series suggest that operative outcomes are comparable amongst different age groups following surgery with curative intent for non-small cell lung cancer (NSCLC). The purpose of this study was to compare morbidity and mortality after NSCLC surgery in older patients (≥ 75 years) versus younger patients (< 75 years) and identify independent predictive risk factors.

**Methods:**

We identified 2015 patients with postoperative stages IA to IIIA according to AJCC/UICC 7th edition who had undergone NSCLC surgery with curative intent at a single specialized lung cancer center from January 2010 to December 2015. A matched-pair analysis was performed on 227 older patients and corresponding 227 younger patients. Short-term surgical outcomes were postoperative morbidity, length of hospital stay, 30-day and 90-day mortality. Long-term operative outcomes were disease-free and overall survival.

**Results:**

454 patients were included in the matched-pair analysis. 36% of younger patients developed postoperative complications versus 42% in older patients (*p* = 0.163). Age was not significantly associated with the occurrence of postoperative complications. Median length of hospital stay was 14 days in older patients and 13 days in younger patients (*p* = 0.185). 90-day mortality was 2.2% in younger patients compared to 4% in older patients (*p* = 0.424). In patients aged 75 and older impaired performance status (ECOG ≥ 1) was associated with decreased overall survival (HR = 2.15, CI 1.34–3.46), as were preoperative serum C-reactive protein / albumin ratio ≥ 0.3 (HR = 1.95, CI 1.23–3.11) and elevated preoperative serum creatinine levels ≥ 1.1 mg/dl (HR = 1.84, CI 1.15–2.95). In the younger cohort male sex (HR = 2.26, CI 1.17–4.36), postoperative stage III disease (HR 4.61, CI 2.23–9.54) and preoperative anemia (hemoglobin < 12 g/dl) (HR 2.09, CI 1.10–3.96) were associated with decreased overall survival.

**Conclusions:**

Lung resection for NSCLC in older patients is associated with postoperative morbidity and mortality comparable to those of younger patients. In older patients, physical activity, comorbidities and nutritional status are related to survival and should influence the indication for surgery rather than age alone.

**Supplementary Information:**

The online version contains supplementary material available at 10.1186/s12957-024-03491-6.

## Introduction

Lung cancer is the most common cause of cancer-related mortality worldwide with a 5-year survival rate of 21.7% [1]. Non-small cell lung cancer (NSCLC) constitutes approximately 85% of all new lung cancer cases. For patients with early-stage NSCLC surgery is the treatment of choice, providing the highest probability of cure [2]. As our population ages, older populations are undergoing major surgery for lung cancer. Comparably, the median age at diagnosis of patients with NSCLC has increased to 71 years in the US [3]. Because older patients often present with numerous comorbidities, which are associated with postoperative morbidity, the decision to perform a lung resection on an older patient may be challanging. Some studies have shown that lobectomy can be perfomed with low mortality and postoperative morbidity in older populations compared with corresponding younger patients [4–8], whereas others report higher postoperative morbidity or mortality in older patients [9, 10]. The conflicting data has led to some uncertainty, which is why clinicians have commenly offered less aggressive treatments, such as sublobar resections, to older patients. Whether the cautious indication for surgery in completely resectable lung cancer is justified on the basis of chronological age remains controversial.

To date, there are no data available from large patient cohorts that controlled for matching factors in the analysis of postoperative morbidity and mortality in different age groups. We performed a matched-pair analysis to determine whether older age is a predictor of postoperative outcomes after surgery for resectable NSCLC, independent of sex, histology, pathological tumour stage and functional status.

## Methods

### Study Design

**Fig. 1:**
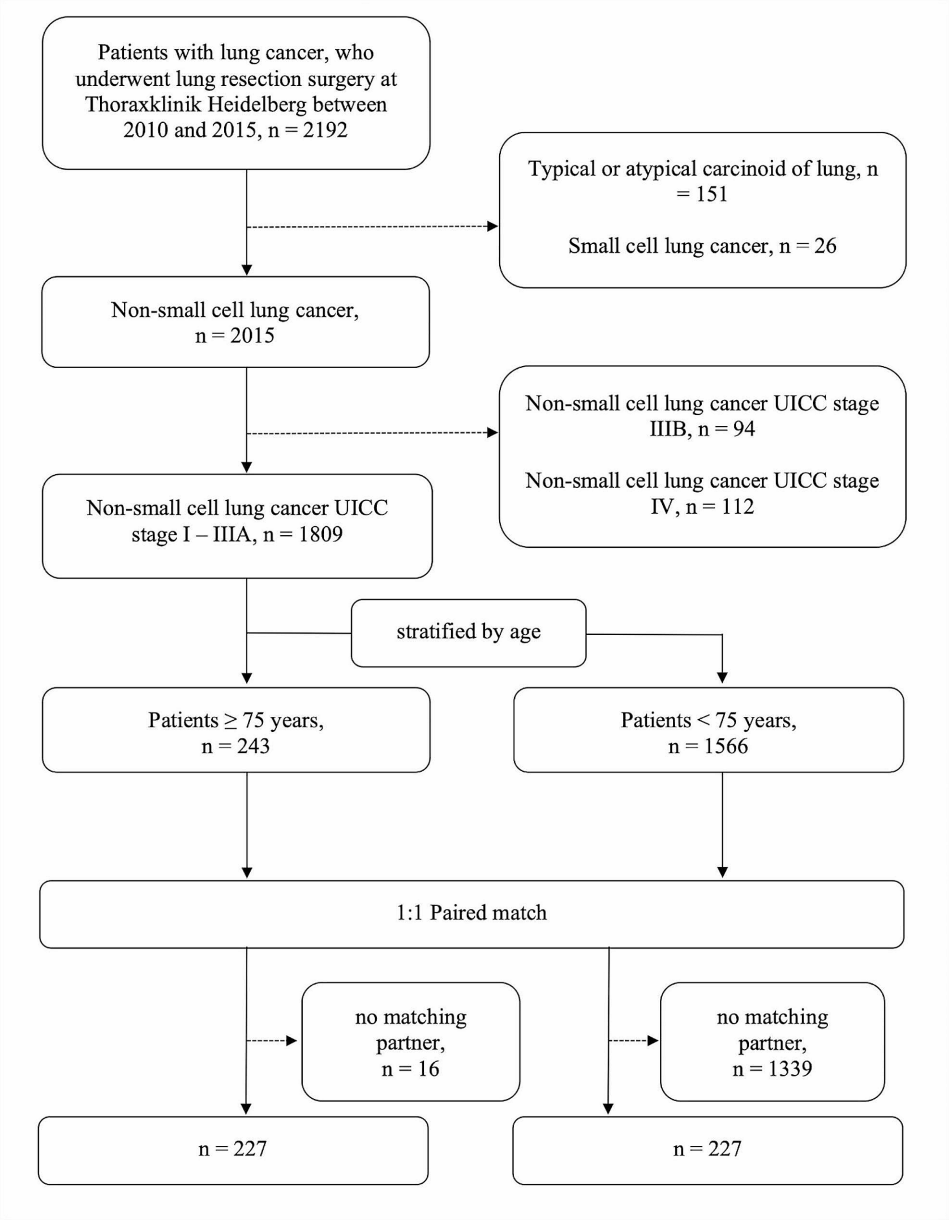
Flow chart of the study population

Patients were identified from our prospectively maintained institutional database at the Department of Thoracic Surgery at Heidelberg University Hospital. All patients receiving medical treatment at the Thoraxklinik of the University Hospital Heidelberg are asked for enrollment in the biomaterial bank, which includes the collection of biomaterials and patient data that are centrally managed in our institutional database. A database query identified 2192 patients who underwent surgery for lung cancer between January 2010 and December 2015. For this analysis, we excluded patients who received surgery for lung cancer entities other than NSCLC and those with stage IIIB or stage IV disease. Furthermore, we performed exact one-to-one case-control matching with age as the defining grouping variable, resulting in two study cohorts of 227 patients each. The matching variables were postoperative tumor stage (stage I vs. stage II vs. stage IIIA), Eastern Cooperative Oncology Group score (ECOG 0 vs. ECOG 1), sex, histological type (squamous cell carcinoma vs. adenocarcinoma vs. other NSCLC type) and year of surgery (2010 vs. 2011 vs. 2012 vs. 2013 vs. 2014 vs. 2015). The enrollment process is illustrated in Fig. 1. This study was performed in line with the principles of the Declaration of Helsinki. Approval was granted by the Ethics Committee of Heidelberg University (Ethics Vote No. 080/2006).

### Assessment of comorbidities

Cardiac comorbidities included myocardial infarction, valvular heart disease, heart failure, cardiomyopathy and atrial fibrillation. Vascular comorbidities included peripheral vascular disease, symptomatic or treated carotid artery stenosis, symptomatic or treated aortic aneurysm, thrombosis, arterial hypertension and pulmonary embolism. Cerebrovascular comorbidities include stroke and transient ischemic attack (TIA). Respiratory comorbidities included COPD, asthma, respiratory insufficiency defined as preoperative capillary oxygen partial pressure < 70 mmHg and / or preoperative capillary carbon dioxide partial pressure > 45 mmHg. Renal insufficiency was defined as glomerular filtration rate < 60 ml / min calculated according to Cockcroft and Gault.

### Surgery and postoperative follow-up

Endobronchial double-lumen intubation allowed single-lung ventilation. Patients underwent either video-assisted thoracoscopic surgery (VATS) with 3 incisions or an anterolateral thoracotomy entering through the fourth or fifth intercostal space. All operatios were completed with an systematic mediastinal and hilar nodal dissection. Postoperative follow-up consisted of chest computed tomography scans every 6 months for the first 2 years and every 12 months thereafter. Tumor recurrence was confirmed by the institutional tumor board. Patients with high-risk stage IB, and stages II and higher, according to AJCC/UICC 7th edition were considered for adjuvant platinum-based chemotherapy. Lung function tests were repeated on all follow-up visits.

### Outcome definition

We choose a matched-pair case-control design to compare baseline variables, comorbidities as well as short-term and long-term surgical outcomes between age groups. As additional risk factors for surgical outcome, we calculated comorbidity scores, i.e. the Charlson comorbidity index (CCI) and Simplified comorbidity score (SCS), as well as inflammation-based scores, i.e. the C-reactive protein/albumin ratio (CAR), Glasgow prognostic score (GPS) and modified Glasgow prognostic score (mGPS). Postoperative morbidity was definited as any of the following: respiratory insufficiency, persistant air leak for more than 3 days, pleural effusion or pneumothorax, bronchial stump insufficiency, postoperative bleeding, acute kidney injury, pneumonia, pleural empyema, surgical site infection or any cardiovascular events. We defined length of hospital stay as time between the day of surgery and the day of hospital discharge. We report postoperative mortality within 30 and 90 days after surgery. Long-term surgical outcomes were disease-free and overall survival. Median follow-up was 38 months. 2.7% of patients were lost to follow-up. Disease-free survival was defined as the date of surgery to the date of locoregional recurrence, distant matastasis or death of any cause. In the disease-free survival analysis we only considered patients with a minimum follow-up of 6 months to exclude patients with occult metastases at the time of surgery. Patients who did not have an event were censored at the time of the last follow-up.

### Statistical analysis

Continous variables are reported as means with standard deviation or median with range or interquartile range. Categorical variables as reported as absolute and relative frequencies. In order to reduce selection bias when comparing different age groups, an exact 1:1 matching model was used. Out of all patients who received surgery with curative intent for stage I to IIIA NSCLC between January 2010 and December 2015, 243 were olden than 75 years at the time of diagnosis. In order to identify a matched group of patients in the younger cohort, 5 covariates were defined: postoperative tumor stage, ECOG score, sex, histological type and year of surgery. To determine the appropriate matching variables, a multivariate Cox proportional-hazards model was computed for the entire study population of patients with stage I to IIIA NSCLC (*n* = 1809) to assess risk factors for overall survival. Variables associated with overall survival included age, postoperative tumor stage, ECOG score, sex, histological type, surgical approach (thoracotomy vs. VATS), and residual tumor status. The surgical approach and residual tumor status strongly correlated with tumor stage and were unevenly distributed, with only 14.1% of patients receiving VATS and residual tumors found in 5.8% of patients. Therefore, these variables were not used as matching variables. To account for differences in follow-up time, the year of surgery was included as a matching variable. To verify the quality of the matching procedure, standardized mean differences were calculated before and after matching. The Cox proportional hazards model for overall survival of the entire study population and the characteristics of younger patients and patients aged 75 and older, including standardized differences before and after matching, are displayed in the supplementary material.

This led to two study groups with 227 patients each. 94.3% of the older patients remained in the analysis, 16 patients could not be matched. Both age groups were compared in terms of demographic data, laboratory values, postoperative complications, length of hospital stay and postoperative mortality using McNemar tests for categorical data and paired t-tests or Wilcoxon-Mann-Whitney tests for continuous data. A logistic regression model was used to identify variables associated with postoperative complications. In order to identify risk factors for the long-term surgical outcomes we applied log-rank tests on preoperative variables on each group seperately and multivariate Cox proportional-hazards models. Since this was a retrospective exploratory data analysis p-values are of descriptive nature. A two-sided p-value of < 0.05 was considered statistically significant. All statistical analyses were performed using SPSS version 25.0 and R version 4.1.1.

## Results

### Patient population

We identified a total of 454 patients who met the inclusion criteria (Fig. 1). The median age in the younger cohort was 65 years (range 20–74) and 78 years (range 75–91) in the older cohort. Table 1 displays clinical data and tumor characteristics. Patients in the older cohort presented with a higher American Society of Anaesthesiology (ASA) Score (*p* = 0.017) and were more likely to undergo video-assisted thoracoscopic surgery (*p* < 0.001). Younger age was associated with a higher rate of pneumonectomies (*p* = 0.048). Leading comorbidities were respiratory (57.3% in the younger cohort vs. 49.3% in the older cohort, *p* = 0.115), vascular (36.1% vs. 24.2%, *p* = 0.008), and cardiac disease (25.6% vs. 27.8%, *p* = 0.657). Younger patients more frequently presented with vascular disease (*p* = 0.008), whereas older patients were more likely to present with chronic kidney disease (11% vs. 44.5%, *p* < 0.001). There were no differences in cardiac, cerebrovascular or metabolic comorbidities between groups. Older patients had a smaller proportion of current smokers (*p* < 0.001). Preoperatively, older patients presented with a higher predicted FEV_1_ (*p* = 0.001). Younger patients were more likely to receive adjuvant chemotherapy (25.1% vs. 11.9%, *p* < 0.001) or adjuvant chemoradiotherapy (26.0% vs. 4.0%, *p* < 0.001).

**Table 1:** Differences in patient characteristics between age groups

	20–74 years (*n* = 227)	75–91 years (*n* = 227)	*p* value
Sex (n, %)			> 0.99
Male	148 (65.2)	148 (65.2)	
Female	79 (34.8)	79 (34.8)	
Postoperative tumor stage (n, %)			0.433
IA	46 (20.3)	39 (17.2)	
IB	57 (25.1)	64 (28.2)	
IIA	27 (11.9)	32 (14.1)	
IIB	31 (13.7)	26 (11.5)	
IIIA	66 (29.1)	66 (29.1)	
Pathological T category (n, %)			0.762
T1	52 (22.9)	41 (18.1)	
T2	112 (49.3)	126 (55.5)	
T3	52 (22.9)	48 (21.1)	
T4	11 (4.8)	12 (5.3)	
Pathological N category (n, %)			0.926
N0	140 (61.7)	141 (62.1)	
N1	45 (19.8)	45 (19.8)	
N2	42 (18.5)	41 (18.1)	
ECOG (n, %)			> 0.99
0	125 (55.1)	125 (55.1)	
1	102 (44.9)	102 (44.9)	
Histologic type (n, %)			0.711
Squamous cell carcinoma	96 (42.3)	96 (42.3)	
Adenocarcinoma	116 (51.1)	116 (51.1)	
Large cell carcinoma	10 (4.4)	6 (2.6)	
Sarcomatoid carcinoma	2 (0.9)	2 (0.9)	
Adenosquamous carcinoma	2 (0.9)	5 (2.2)	
Not otherwise specified	1 (0.4)	2 (0.9)	
Adenocarcinoma subtype (n, %)			
lepidic	15 (6.6)	18 (7.9)	0.690
papillary, acinar	54 (23.8)	22 (9.7)	0.053
solid, micropapillary	42 (18.5)	74 (32.6)	**0.013**
Surgery (n, %)			**< 0.001**
Thoracotomy	224 (98.7)	188 (82.8)	
VATS	3 (1.3)	39 (17.2)	
Type of resection (n, %)			**0.048**
Wedge resection	13 (5.7)	15 (6.6)	
Segmentectomy	13 (5.7)	14 (6.2)	
Lobectomy	163 (71.8)	183 (80.6)	
Bilobectomy	7 (3.1)	5 (2.2)	
Pneumonectomy	31 (13.7)	10 (4.4)	
Adjuvant therapy (n, %)			
Chemotherapy	57 (25.1)	27 (11.9)	**< 0.001**
Radiotherapy	10 (4.4)	18 (7.9)	0.186
Combined	59 (26.0)	9 (4.0)	**< 0.001**
No adjuvant therapy	101 (44.5)	173 (76.2)	**< 0.001**
ASA classification (n, %)			**0.017**
1 + 2	46 (20.3)	26 (11.5)	
3 + 4	181 (79.7)	201 (88.5)	
Smoking status (n, %)			**< 0.001**
Current	51 (22.5)	18 (7.9)	
Former	66 (29.1)	18 (7.9)	
Never	110 (48.5)	191 (84.1)	
CCI (median, range)	3 (2–7)	3 (2–7)	0.056
SCS (median, range)	8 (1–19)	6 (1–18)	**< 0.001**
Vascular disease (n, %)	82 (36.1)	55 (22.4)	**0.008**
Chronic kidney disease (n, %)	25 (11.0)	101 (44.5)	**< 0.001**
FEV_1_ (% predicted, mean)	77.5 ± 21.6	84.1 ±19.9	**< 0.001**
FVC (% predicted, mean)	87.0 ± 16.8	91.3 ±15.5	**0.003**
FEV_1_/FVC ratio (mean)	68.3 ± 11.7	69.3 ± 11.6	0.258

Values are presented as either percent, mean ± standard deviation or median (range), as indicated. ASA, American Society of Anesthesiologists; CCI, Charlson comorbidity index; ECOG, Eastern cooperative oncology group; FEV1, Forced expiratory volume in the first second; FVC, Forced vital capacity; SCS, Simplified comorbidity score; VATS, Video-assisted thoracoscopic surgery

### Short-term surgical outcomes

30- and 90-day mortality rates in younger patients were 1.8% and 2.2% compared with 1.8% and 4.0%, respectively in older patients. The 90-day mortality in younger patients did not differ significantly from that in patients aged 75 and older (*p* = 0.424). All cases of 30-day mortality were in-hospital deaths. The causes for in-hospital mortality in older patients (*n* = 4) were arrhythmia, pneumonia, congestive heart failue and pleura empyema. The causes for in-hospital mortality in the younger cohort (*n* = 4) were myocardial infarction, pneumonia, bronchial stump insufficiency and stroke. All younger patients who died in hospital underwent a pneumonectomy. 36% of the younger patients developed at least one postoperative complication versus 42% in older patients (*p* = 0.163). Overall, leading postoperative complications were symptomatic arrhythmia, pneumonia, respiratory failure, persistent air leak, pleural effusion and pneumothorax. The frequencies of postoperative complications between both cohorts revealed no significant differences and are demonstrated in Table 2. A total of 15 older patients developed a complication that required surgical intervention. The reasons for surgical interventions were pneumothorax or pleural effusion (*n* = 7), prolonged air leak (*n* = 4), postoperative bleeding (*n* = 2), pleural empyema (*n* = 2), respiratory insufficiency (*n* = 1) and bronchial stump insufficiency (*n* = 1). The reasons for surgical revision in 25 younger patients were pneumothorax or pleural effusion (*n* = 13), bronchial stump insufficiency (*n* = 6), persistent air leak (*n* = 3), postoperative bleeding (*n* = 3), pleural empyema (*n* = 2), surgical site infection (*n* = 2) and other surgical complications (*n* = 2). The occurence of one or more postoperative complications was associated with reduced overall survival, both in the entire study cohort (*n* = 454, *p* = 0.001) as well as in the older (*p* = 0.031) and younger cohort seperately (*p* = 0.014). The causes of postoperative readmission to intensive care in older patients were arrhythmia (*n* = 7), pneumonia (*n* = 4), congestive heart failue (*n* = 1), respiratory insufficiency (*n* = 1), gastrointestinal bleeding (*n* = 1) and delirium (*n* = 2) compared to arrythmia (*n* = 6), pneumonia (*n* = 2), stroke (*n* = 1), and gastrointestinal bleedung (*n* = 1) in the younger cohort. The extend of surgical resection was significantly associated with the occurrence of postoperative complications (lobectomy or bilobectomy: OR 5.28, CI 2.32–13.41 and pneumonectomy: OR 3,50, CI 1.17–11.09). The likelihood of postoperative complications was lower in females (OR 0,57, CI 0.35–0.92) as shown in Table 3. Also per 10% increase in FEV_1_ the likelihood of postoperative complications was reduced with an OR of 0.86 (CI 0.76–0.97). Chronological age was not significantly associated with the occurrence of postoperative complications. The median length of hospital stay in the younger cohort was 13 days (range 4–58) compared to 14 days (range 5–72) in the older cohort (*p* = 0.185).

**Table 2:** Differences in postoperative complications between age groups

	20–74 years (*n* = 227)	75–91 years (*n* = 227)	*p* value
Patients with postoperative complications			0.163
0	146 (64.3)	131 (57.7)	
≥ 1	81 (35.7)	96 (42.3)	
Return to OR	25 (11.5)	15 (6.6)	0.123
Return to ICU	11 (4.8)	16 (7.0)	0.424
Reintubation	4 (1.8)	4 (1.8)	> 0.99
Cardiovascular complications	31 (13.7)	37 (16.3)	0.504
Symptomatic arrhythmia	27 (11.9)	30 (13.2)	
Myocardial infarction	5 (2.2)	3 (1.3)	
Congestive heart failure	1 (0.4)	4 (1.8)	
Cardiac arrest (non-lethal)	2 (0.9)	1 (0.4)	
Stroke	2 (0.9)	0	
Thrombosis	0	1 (0.4)	
Infectious complications	35 (15.4)	30 (9.2)	0.596
Pneumonia	28 (12.3)	25 [11]	
Pleural empyema	2 (0.9)	1 (0.4)	
Surgical Site Infection	6 (2.6)	3 (1.3)	
Sepsis	1 (0.4)	2 (0.9)	
Respiratory complications	32 (14.1)	34 (15.0)	0.892
Respiratory failure	11 (4.8)	9 (4.0)	
Persistant air leak (> 3 days)	9 (4.0)	15 (6.6)	
Pneumothorax or pleural effusion	12 (5.3)	9 (4.0)	
Bronchial stump insufficiency	5 (2.2)	1 (0.4)	
Other	10 (4.4)	17 (7.5)	0.248
Postoperatvie bleeding	5 (2.2)	5 (2.2)	
Acute kidney injury	2 (0.9)	5 (2.2)	
Postoperative delirium	4 (1.8)	7 (3.1)	

Values are presented as absolute and relative frequencies. OR, operating room; ICU, intensive care unit.

**Table 3:** Variables associated with postoperative complications

	Odds Ratio	Lower CI	Upper CI	*p*-value
Age > 75	1.62	0.97	2.70	0.067
FEV1 predicted per 10% increase	0.86	0.76	0.92	**0.016**
CCI > median	0.94	0.51	1.73	0.849
SCS > median	1.56	0.96	2.57	0.075
CAR ≥ 0.3	1.10	0.44	2.81	0.846
Female sex	0.57	0.35	0.92	**0.023**
Stage II vs. Stage I	1.38	0.80	2.38	0.24
Stage III vs. Stage 1	1.58	0.89	2.80	0.118
ECOG ≥ 1	1.49	0.94	2.37	0.087
ASA 3 + 4	1.37	0.74	2.60	0.332
VATS vs. thoracotomy	0.66	0.29	1.43	0.298
BMI ≥ 30	1.12	0.66	1.88	0.684
Cardiac disease	0.75	0.44	1.26	0.275
Vascular disease	1.08	0.66	1.74	0.763
Cerebrovascular disease	0.90	0.37	2.13	0.807
Respiratory disease	1.10	0.69	1.75	0.701
Chronic kidney disease	1.22	0.72	2.07	0.464
Diabetes	0.81	0.44	1.49	0.499
GPS ≥ 1	0.72	0.32	1.56	0.414
Bi-/lobecomy vs. sublobar resection	5.28	2.32	13.41	**< 0.001**
Pneumonectomy vs. sublobar resection	3.50	1.17	11.09	**0.028**

ASA, American Society of Anesthesiologists, CCI Charlson comorbidity index, CI, confidence interval; ECOG, Eastern cooperative oncology group; FEV_1_ Forced expiratory volume in the first second; FVC Forced vital capacity, GPS, Glasgow prognostic score; SCS, Simplified comorbidity score; VATS, Video-assisted thoracoscopic surgery

### Univariate analysis of long-term surgical outcomes

**Fig. 2:**
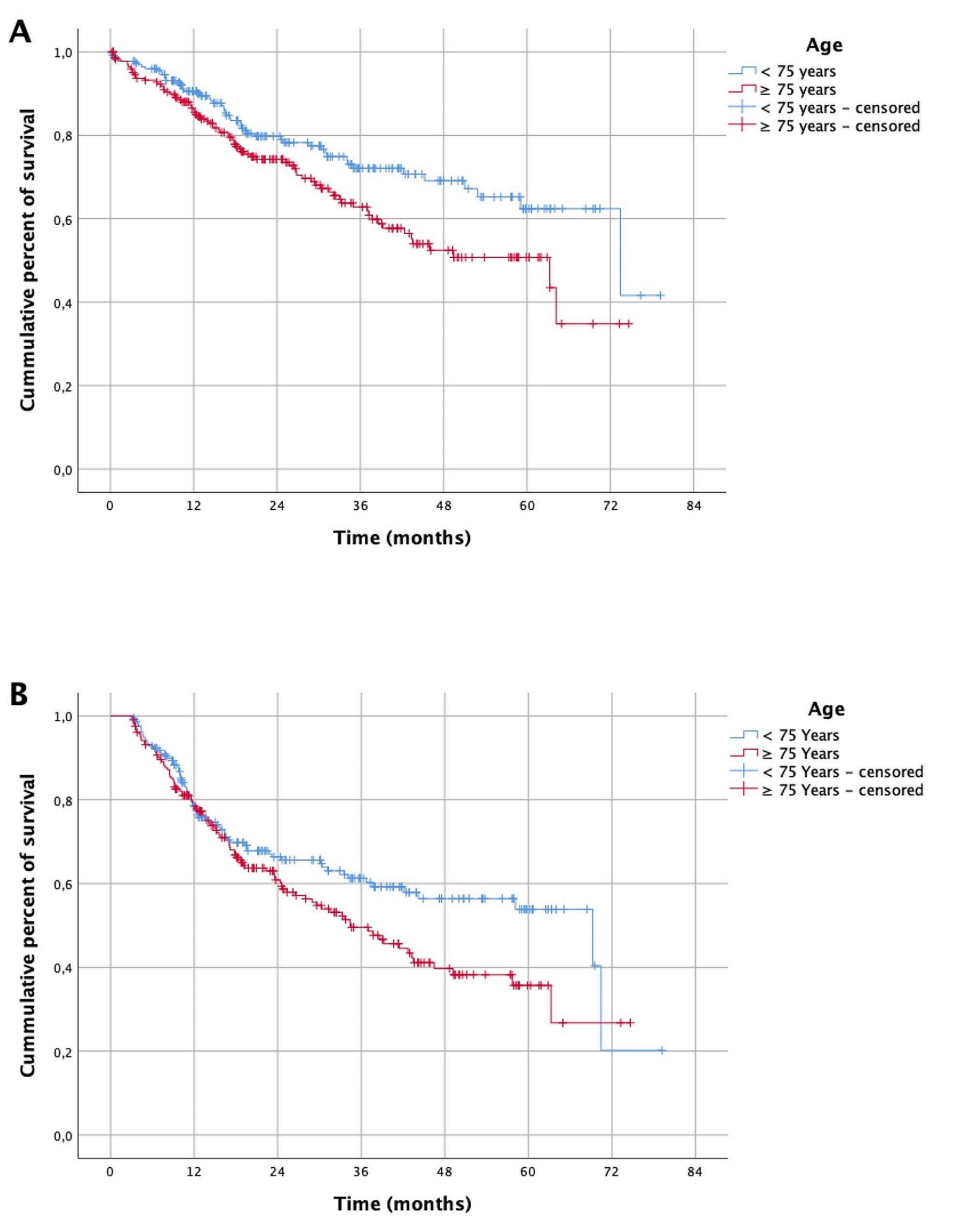
Overall survival (**A**) and disease-free survival (**B**) of the matched-pair groups

Median overall survival in the entire cohort was 64.2 months (CI 55.7–72.5). Older patients showed a median overall survival of 63.3 months (CI 37.1–89.3) with 1, 3 and 5 year overall survival of 86%, 62% and 51%, respectively. In the younger patients median overall survival was 73.4 months (CI 50–96.9) with 1, 3 and 5 year overall survival of 91%, 72% and 62%, respectively (*p* = 0.015).The median disease-free survival was 43.5 months (CI 31.7–55.3) in older patients vs. 69.3 months (CI 31.9–106.7) in the younger cohort (Fig. 2) (*p* = 0.037). The overall (OS) and disease-free survival (DFS) depending on different prognostic factors are displayed in the supplementary material. Variables that were associated with reduced OS in patients aged 75 and older were male sex, ECOG score ≥ 1, Simplified Comorbidity Score (SCS) ≥ median, high C-reactive protein/albumin ratio (CAR) and elevated serum creatinine (for all *p* < 0.01). In contrast, in the younger patients OS was not significantly affected by comorbidities, comorbidity indices and inflammation-based scores, such as the CAR, GPS and mGPS. The extend of resection was significantly associated with OS in younger patients (*p* = 0.047), however, we observed a strong correlation with pathologic stage, as patients with sublobar resection presented with an early stage of diesease. Only in younger patients the predominant histologic subtype in lung adenocarcinoma was associated with survival with lepidic adenocarcinoma showing the best and micropapillary and solid adenocarcinoma showing the worst OS (OS, *p* = 0.01; DFS, *p* = 0.028).

### Multivariate analysis of long-term surgical outcomes

In younger patients male sex (HR 2.26, CI 1.17–4.36), advanced stage III disease (HR 4.61, CI 2.23–9.54) and preoperative anemia (hemoglobin < 12 g/dl) (HR 2.09, CI 1.10–3.96) were significantly associated with OS in the multivariate analysis. In older patients ECOG ≥ 1 (HR = 2.15, CI 1.34–3.46), CAR ≥ 0.3 (HR 1.95, CI 1.23–3.11) and elevated preoperative serum creatinine levels (HR 1.84, CI 1.15–2.95) were associated with a reduced OS. Postoperative tumor stage (stage III vs. stage I and stage II vs. stage I) was significantly associated with DFS in in both cohorts. In the younger cohort elevated preoperative serum creatinine (HR 1.82, CI 1.09–3.05) was associated with lower DFS. In older patients male sex (HR 1.62, CI 1.02–2.58), ECOG score ≥ 1 (HR 1.64, CI 1.09–2.47), diabetes (HR 1.89, CI 1.17–3.08) and obesity, defined as BMI ≥ 30, (HR 1.72, CI 1.07–2.77) was associated with DFS.

## Discussion

Non-small cell lung cancer is a disease of older people. In this large matched-pair study, age was not associated with either postoperative survival or the occurrence of postoperative complications after surgery for resectable NSCLC. We demonstrate that the postoperative 30-day mortality was consistent at 1.8% across both age groups, whereas the 90-day mortality exhibited a non-significant difference of 2.2% in patients < 75 years compared to 4.0% in the older age group. Similar findings were reported in the studies by Rivera et al. and Park et al., who indicated an increase in 90-day mortality compared to 30-day mortality in older study populations, while the increase was relatively smaller in younger patients [11, 12]. Several publications have reported varied 30-day mortality rates following lung resection in patients with NSCLC, ranging from 0 to 9% [13–21]. The reported 90-day mortality rates ranged from 1.5 to 10% [5, 11, 15, 21–23]. These variations in mortality data may be attributed to the heterogeneity of the study populations. Notably, some studies highlighted that older patients exhibited higher perioperative mortality compared to younger cohorts, emphasizing the impact of age on surgical outcomes [12, 20, 24]. Rivera et al. demonstrated a significantly higher 30- and 90-day mortality in patients aged above 70 in a matched study including 1969 patients [12]. However, only patients with NSCLC stage I and II were included in the analysis and tumor stage as a confounder for perioperative mortality and overall survival was not chosen as a matching variable.

In this study 42% of patients ≥ 75 years developed at least one postoperative complication, compared to 36% in the younger patients. Notably, the literature reports varied rates of postoperative complications after lung resection in NSCLC, ranging from 8.4 to 66.7% [13–21, 23, 25]. The wide range of postoperative complication rates probably results from differences in the definition of complications, follow-up period and patient selection. Some publications indicated no significant difference in postoperative complication rates between younger and older patient populations, while others emphasized a substantial disparity in complication rates depending on patient age [5, 14, 15, 26, 27]. Park et al. compared the postoperative complication rate of 285 patients ≥ 70 years versus 1055 patients < 70 years with stage I NSCLC and demonstrated a significantly higher rate in older patients [11]. However, differences in patient populations, particularly with respect to T category, sex and surgical approach, may have biased the conclusions. We demonstrate an association between male sex, loss of FEV_1_, extended surgical resections and the development of postoperative complications. Similarly, Saji et al. demonstrated a 2.7-fold increased risk of developing postoperative complications in men [28]. In line with the works of Kutluk et al. and Sezen et al., arrhythmias were the leading postoperative complication, regardless of age [23, 29]. Others such as Amer et al. described a significantly higher rate of postoperative arrhythmias and need for intensive care in patients over 80 years of age [14]. In this work there was no association between the surgical approach and the occurrence of postoperative complications. However, only 9% of patients underwent video-assisted thoracoscopic surgery, and these had a lower tumor stage and were older which reflects our clinical practice and that of others [30, 31].

There was no difference in the length of hospital stay between the groups. In the literature reported hospital stay after lung resection surgery varies between 4 and 21 days [31]. However, differences across healthcare systems, where treatments and follow-ups are shifted to outpatient and post-hospital settings make a comparison difficult.

Nearly twice as many patients in the younger cohort received adjuvant chemotherapy compared to the older cohort. Overall, the majority of patients aged ≥ 75 years did not receive guideline-recommended adjuvant therapy, a practice that has been described by others and is certainly due to the lack of data on adjuvant chemotherapy in patients over 75 years of age. Baldvinsson et al. described that of all patients who underwent lung resection with curative intend in Iceland between 1991 and 2014, only 5% in the group of patients ≥ 75 years received adjuvant chemotherapy [15]. Similarly, in a study of 337 patients ≥ 80 years with stage I to IIIA NSCLC, only 5 patients received adjuvant chemotherapy and 1 patient received adjuvant radiotherapy following lung cancer surgery [21]. Yamanashi et al. compared 246 patients ≥ 75 years with stage IB - IIIA NSCLC who received chemotherapy (*n* = 102) or best supportive care (*n* = 144) in a retrospective observational study. After controlling for baseline characteristics using propensity score matching, they found adjuvant chemotherapy being significantly associated with a reduced disease-free survival [32]. In contrast, a meta-analysis including 4584 patients with stage I – IIIA NSCLC demonstrated a positive effect of adjuvant chemotherapy on disease-related survival in patients ≥ 70 years of age compared to younger cohorts [33]. Similarly, Yano et at. showed that adjuvant postoperative chemotherapy did not adversely affect the clinical performance status 2 years after treatment in a prospective study of 272 patients with NSCLC aged ≥ 75 years [34]. While randomized controlled trials of adjuvant chemotherapy in older patients are lacking, the importance of isolated adjuvant chemotherapy is decreasing due to the perioperative use of molecular targeted therapy and immunotherapy.

Overall, the median OS was 64.2 months (95% CI 55.7–72.5). Both overall survival and DFS were higher in the younger patients. Median OS was 63.3 months and DFS 43.5 months in the older patients, while median OS was 73.4 months and DFS 69.3 months in the younger patients. In both groups, a significant association was found between OS and ECOG status, sex, and postoperative tumor stage. The influence of tumor stage on OS after lung resection procedures in NSCLC has been demonstrated in many studies. The Kaplan-Meier graphs (Fig. 2) show a difference in OS in patients aged ≥ 75 years probably because of a higher likelihood of dying from non-tumor-related causes as we do not demonstrate lung cancer specific survival. In older patients, a more pronounced association between existing comorbidities and survival probability was demonstrated compared to the younger patient group. Patients with diabetes showed a significantly reduced OS and DFS in the univariate analysis and patients with a preoperative serum creatinine ≥ 1.1 mg/dl had reduced OS in the univariate and multivariate regression analysis. Inflammation-based scores showed an association between age and survival. In patients aged ≥ 75 years, SCS > median, CAR ≥ 0.3, GPS ≥ 1, and mGPS ≥ 1 were all significantly associated with reduced OS in univariate analyses. The association between CAR and OS remained in the multivariate regression analysis. In younger patients there was no significant association between inflammation-based scores and survival. In line with these results Hino et al. demonstrated in octogenarians that GPS ≥ 1 and CCS ≥ 2 was associated with reduced OS [21]. Miyazaki et al. also reported an association of GPS and OS in a study population of 97 patients aged ≥ 80 with NSCLC stage I [35]. Miura et al. demonstrated that sarcopenia that progresses in the postoperative course is associated with reduced OS in older patients with NSCLC [36]. Whether this association in the older patients is an expression of a reduced nutritional status or an increased catabolic situation due to the tumor disease remains controversial. On examining the histological subtypes, we demonstrated that adenocarcinomas were significantly less frequent in older patients, whereas there were no age-related differences in the frequency of large cell, adenosquamous, and sarcomatoid carcinomas. This finding is consistent with a study by Mery et al., who demonstrated that squamous cell carcinomas were more frequently observed in patients aged ≥ 75 years compared to younger populations [37]. Only in the younger cohort, a significant association between adenocarcinoma subtypes and overall survival was observed with lepidic adenocarcinoma being associated with the most favorable outcome, which is consistent with findings from the literature [38–40]. It is likely that the generally poorer OS in older patients does not reflect the beneficial effect of the subtype.

International guidelines stress the role of stereotactic radiotherapy in older patents with early stage NSCLC or patients deemed inoperable due to comorbidities or limited functional status [41]. Haasbeck et al. could demonstrate that the introduction of stereotactic radiotherapy led to a reduction of the number of untreated patients [42]. Our findings suggest that surgical options remain viable and potentially beneficial for selected older patients. This perspective complements the ESMO guideline by emphasizing that a tailored approach should be adopted, where the choice between surgery and stereotactic radiotherapy is made based on a comprehensive assessment of the patient’s performance status, comorbidities, and personal preferences, rather than age alone. The assessment might include a stair-climbing test, which has been shown to predict non-cancer-specific and overall-survival in a cohort of 283 patients undergoing VATS for stage I to IV NSCLC [43]. Preoperative assessment, with a focus on older patients, might include the Short Nutritional Assessment Questionnaire, the Short Physical Performance Battery, and frailty scores such as the Groningen Frailty Index or the Geriatric-8 score, which have been shown to be associated with postoperative complications in patients 70 years of age and older with stage I or II NSCLC [44].

This study has some limitations. Firstly, the retrospective design, leading to incomplete data sets. Secondly, patients receiving VATS had a lower tumor stage and were older, which constitutes a selection bias for the comparison of surgical approaches and postoperative complications. The aim of matching is to reduce bias between study cohorts. However, the number of possible matching variables is limited by the number of patients. A limitation of all matching methods is that the influence of confounders cannot be ruled out. Thirdly, as this was a single-center study, the results are limited by potential bias. The high rate of pneumonectomies could be considered an institutional bias. Another limitation of this study is that it examined patients who underwent NSCLC surgery between January 2010 and December 2015, using the 7th edition of the AJCC/UICC classification system. Due to the older patient population, the thoracotomy rate was high and the length of hospital stay was longer than what would be expected in a population undergoing VATS.

## Conclusion

In summary, we could demonstrate that lung resection with curative intent in older patients with NSCLC can be performed safely, with postoperative morbidity comparable to that of younger patient groups. No significant differences in postoperative complications, length of hospital stay and perioperative mortality could be shown between the matched-pair groups. Contrary to what one might assume, age was not a predictor of the occurance of postoperative complications. Also the rate of postoperative reintubation, return to operation room, need for intensive care, cardiovascular events, pneumonia or bronchopleural fistula showed no age-related differences. Differences in overall survival between age groups occurred later in the postoperative course, most likely due to non tumor-related mortality. It appears that, particularly in older patients, physical activity, comorbidities and nutritional status are related to survival and should influence the indication for surgery rather than age alone.

### Electronic supplementary material

Below is the link to the electronic supplementary material.

Supplementary Material 1

## Data Availability

The datasets used and/or analyzed during the current study are available from the corresponding author on reasonable request.

## Abbreviations

ASA: American Society of Anesthesiology
CAR: C-reactive protein/albumin ratio
CCI: Charlson comorbidity index
COPD: Chronic obstructive pulmonary disease
DFS: Disease-free survival
ECOG: Eastern cooperative oncology group
FEV_1_: Forced expiratory volume in the first second
FVC: Forced vital capacity
GPS: Glasgow prognostic score
NSCLC: Non-small cell lung cancer
OS: Overall survival
SCS: Simplified comorbidity score
TIA: Transient ischemic attack
VATS: Video-assisted thoracoscopic surgery
