# Supplementary material for: Peri- and postoperative morbidity and mortality in older patients with non-small cell lung cancer: a matched-pair study
Source: World J Surg Oncol. 2024 Aug 8;22:213. doi: 10.1186/s12957-024-03491-6 (PMC11311962; doi:10.1186/s12957-024-03491-6)
Supplement: Supplementary file 1 — Supplementary Material 1 [file 12957_2024_3491_MOESM1_ESM.pdf]

Supplemenary material

Univariate Analysis of Overall Survival and Disease-free Survival

**Variable**	**Overall Survival (OS)**				**Disease-free Survival (DFS)**			
Age, years	**26 - 74 years**		**75 - 90 years**		**26 – 74 years**		**75 – 90 years**	
	**3y OS (%)**	***p*-value**	**3y OS (%)**	***p*-value**	**3y DFS (%)**	***p*-value**	**3y DFS (%)**	***p* value**
Sex		0.015		0.003		0.262		0.007
Male	67.1		55.6		59.9		44.2	
Female	81		76.6		65.2		61.8	
Postoperative tumor stage		< 0.001		0.017		< 0.001		0.001
I	86.3		78.3		79.5		66.6	
II	73.7		50.5		62.7		35.6	
III	43.4		46.5		29.9		33.7	
Histology		0.088		0.083		0.678		0.047
Squamous cell	68.7		63.8		57.5		40.2	
Adenocarcinoma	78.1		66.2		56.3		22.1	
Large cell carcinoma	50.0		44.4		23.8		33.3	
Other	60.0		41.5		37.5		65.6	
Adenocarcinoma subtype		0.010		0.313		0.028		0.462
G1 (lepidic)	no event		74.8		no event		59.2	
G2 (papillary, acinar)	85.9		68.8		61.6		56.6	
G3 (solid, mikropapillary)	66		55.8		56		41.6	
ECOG		0.039		< 0.001		0.025		0.008
0	77.8		74.4		71.4		59	
≥ 1	63.1		43.6		48.3		35.3	
Resection		0.047		0.115		0.872		0.592
Lobar	70.2		61		54.6		27.8	
Sublobar	93.3		89.1		41		30.4	
ASA classification		0.145		0.907		0.859		0.700
1+2	79.2		65.4		65.2		58.6	
3+4	70.2		62.4		61.1		49.2	
CCI		0.236		0.136		0.035		0.338
≤ Median	76.3		68.1		68.3		54.4	
> Median	61.8		52.8		47.9		42.2	
SCS		0.774		0.003		0.209		0.029
≤ Median	73.3		69.0		59.1		55.7	
> Median	72.5		46.7		63.9		37.1	
CAR		0.056		0.006		0.048		0.185
< 0.3	74.1		69.0		65.0		52.9	
≥ 0.3	66.0		46.0		50.1		41.6	
CRP		0.039		0.050		0.013		0.746
< 5mg/dl	78.1		73.4		72.9		52.2	
≥ 5mg/dl	67.6		57.7		54.9		49.8	
GPS		0.092		0.020		0.023		0.277
0	74		67.9		66.8		53.3	
≥ 1	67.4		50.9		49.9		41.9	
modified GPS		0.092		0.019		0.023		0.427

0	74		67.9		66.8		52.5	
≥ 1	67.4		49.9		49.9		43.3	
Preoperative serum creatinine		0.100		0.003		0.005		0.096
< 1.1 mg/dl	74.8		69.5		66.6		55.5	
≥ 1.1 mg/dl	59.4		44.1		40.2		32.3	
Preoperative hemoglobin		0.002		0.028		0.031		0.030
< 12 g/dl	44.8		42.9		41.2		34.2	
≥ 12 g/dl	77.4		67.6		65.6		54	
Body mass index		1		0.925		0.629		0.020
< 30	72.3		64		61.9		54.2	
≥ 30	70.2		55.1		62.3		34.9	

ASA, American Society of Anesthesiologists; CAR, c-reactive protein/albumin ratio; CCI, Charlson comorbidity index; CRP, c-reactive protein; ECOG, Eastern cooperative oncology group; GPS, Glasgow prognostic score; INR, international normalized ratio; p, p-value of the log-rank test; SCS, simplified comorbidity score; UICC, Union internationale contre le cancer; WBC, White blood count

Multivarate Cox proportional hazards model for the overall survival of the entire study population with stage I to IIIA NSCLC

Variable	HR	95% CI		*p* value
Age < 75 (Ref.)				
Age ≥ 75	1.49	1.17	1.89	**< 0.001**
Stage I (Ref.)				
Stage II	1.54	1.19	2.01	**< 0.001**
Stage III	3.11	2.47	3.92	**< 0.001**
ECOG 0 (Ref.)				
ECOG 1	1.61	1.32	1.97	**< 0.001**
ECOG ≥ 2	3.71	2.07	6.67	**< 0.001**
Female (Ref.)				
Male	1.69	1.38	2.07	**< 0.001**
Adenocarcinoma (Ref.)				
Squamous cell carcinoma	0.84	0.69	1.02	0.085
Other	1.35	1.04	1.76	**0.026**
VATS (Ref.)				
Thoracotomy	1.73	1.21	2.46	**0.002**
Residual tumor stage R0 (Ref.)				
Residual tumor stage R≥1	2.301	1.755	3.016	**< 0.001**

CI, confidence interval; ECOG. Eastern cooperative oncology group; HR, hazard ratio; Ref. reference; VATS. Video-assisted thoracoscopic surgery;

Characteristics of the study population stratified by age and standardized mean differences

Variable	20-74 years (n=1566)	75-91 years (n=243)	*p* value	Pre-match SMD	Post-match SMD
Sex			0.362		
Male	964 (61.6)	157 (64.6)		-0.045	< 0.001
Female	602 (38.4)	86 (35.4)		0.045	< 0.001
Histologic type			**0.029**		
Adenocarcinoma	910 (58.1)	119 (49.0)		0.130	< 0.001
Squamous cell carcinoma	500 (31.9)	103 (42.4)		-0.154	< 0.001
Large cell carcinoma	91 (5.8)	9 (3.7)		0.032	< 0.001
Adenosquamous carcinoma	30 (1.9)	7 (2.9)			
Sarcomatoid carcinoma	15 (1.0)	3 (1.2)			
Primary salivary gland-type lung cancer	8 (0.5)	0 (0.0)			
Not otherwise specified	12 (0.8)	2 (0.8)			
Postoperative tumor stage			0.070		
I	591 (37.7)	110 (45.3)		-0.062	0.004
II	430 (27.5)	62 (25.5)		0.021	< 0.001
III	545 (34.8)	71 (29.2)		0.085	< 0.001
Pathological T category			**0.031**		
T1	377 (24.1)	46 (18.9)		0.089	0.085
T2	689 (44.0)	133 (54.7)		-0.153	-0.087
T3	387 (24.7)	52 (21.4)		0.056	0.047
T4	113 (7.2)	12 (4.9)		0.067	-0.014
Pathological N category			0.271		
N0	883 (56.4)	150 (61.7)		-0.077	-0.006
N1	344 (22.0)	49 (20.2)		0.031	< 0.001
N2	339 (21.6)	44 (18.1)		0.063	0.008
ECOG			**< 0.001**		
0	1222 (78.0)	127 (52.3)		0.397	< 0.001
1	329 (21.0)	113 (46.5)		-0.397	< 0.001
≥ 2	15 (1.0)	3 (1.2)			
Surgery			0.314		
VATS	211 (13.5)	41 (16.9)		-0.067	-0.402
Thoracotomy	1353 (86.4)	202 (83.1)		0.064	0.402
Other	2 (0.1)	0 (0.0)			
Residual tumor stage			0.339		
R0	1467 (93.7)	234 (96.3)		-0.085	0.102
R1	77 (4.9)	6 (2.5)		0.079	-0.102
R2	19 (1.2)	3 (1.2)			

Unknown	3 (0.2)	0 (0.0)			
Type of resection			**< 0.001**		
Wedge resection	73 (4.7)	16 (6.6)			
Segmentectomy	38 (2.4)	17 (7.0)		-0.059	-0.026
Lobectomy	1188 (75.9)	195 (80.2)		-0.153	-0.013
Bilobectomy	66 (4.2)	5 (2.1)		-0.075	-0.147
Pneumonectomy	201 (12.8)	10 (4.1)		0.088	0,039

Values are presented as absolute and relative frequencies, ECOG, Eastern cooperative oncology group; SMD, standardized mean difference; VATS, Video-assisted thoracoscopic surgery
